# Urine Screening and 9 Years’ Medical Record System Follow-Up Among School Students in Wenzhou, China

**DOI:** 10.3389/fped.2022.862029

**Published:** 2022-04-25

**Authors:** Tingting Chen, Qing Yang, Hong Xu, Yinv Gong, Xiaoling Guo, Hongzhou Lin, Jianhuan Yang, Jieqiu Zhuang, Junwei Lan, Maoping Chu, Dexuan Wang

**Affiliations:** ^1^Department of Pediatrics, The Second Affiliated Hospital and Yuying Children’s Hospital of Wenzhou Medical University, Wenzhou, China; ^2^Basic Medical Research Center, The Second Affiliated Hospital and Yuying Children’s Hospital of Wenzhou Medical University, Wenzhou, China; ^3^Department of Nephrology, Children’s Hospital of Fudan University, Shanghai, China; ^4^Key Laboratory of Children Genitourinary Diseases of Wenzhou, The Second Affiliated Hospital and Yuying Children’s Hospital of Wenzhou Medical University, Wenzhou, China

**Keywords:** urinary screening, children, dipstick urine analysis, hematuria, proteinuria

## Abstract

School urinary screening programming can be useful for the early detection of renal and urinary disorders. However, urine screening is not included in the school health check-up in our region. Therefore, from February 2012 to March 2021, 12,497 school students were screened for urinalysis, and a long-term follow-up took place *via* an electronic medical record system. Among these screened students, 719 (5.75%) positive individuals received a repeat urinalysis 2 weeks later. During the 9-year medical record system follow-up period, 5 children had renal biopsies and 2 children had a diagnosis of IgA nephropathy (IgAN), while the remaining 3 children were diagnosed with thin basement membrane disease (TBM), primary nephrotic syndrome (PNS), and were suspected of C3 glomerulopathy, respectively. By this, calling for the school urine screening program as a physical examination item for primary and secondary school-aged students will contribute to enabling early detection of urine abnormalities and allow for early treatment.

## Introduction

The clinical manifestations of children with chronic kidney disease (CKD) often lack specificity, which makes it difficult for early diagnosis. Some countries and districts had performed long-term urine screening programs at schools for early detection of kidney disease. However, urine screening is not included in the school health check-up in our region, so this results in missed opportunities for early detection. We, therefore, conducted this study to gain further insight into the abnormal urinalysis in primary and secondary school students in Wenzhou, China. Besides, the feasibility of a school urine screening program as a physical examination item for primary and secondary school-aged students, screening modalities were explored.

## Subjects and Methods

The study protocol was approved by the Institutional Review Board of Children’s Hospital of Fudan University (study number 2012–174). All parents or legal guardians of the included children gave their written consent to participate in the study.

### Subjects

From February 2012 to March 2021, a total of 12,497 school children including 6,550 primary school students (6–7 years old) and 5,947 secondary school students (12–13 years old) from Pingyang, Cangnan, and Yongjia in Wenzhou, China, participated in a mass school urine screening program. The exclusion criteria are as follows: (1) Children who had kidney disease previously or other kidney-related diseases before this screening were excluded. (2) Children with leukocyturia were excluded.

### Methods

The guardians of these children were informed about the importance of urine screening, and informed consent was obtained before screening procedures. The children were asked to empty their bladder at night and midstream urine samples were collected at school. To keep samples fresh, the medical staff tested the urine samples within 1 h qualified in a standardized manner from the local county hospitals. The urine samples were collected twice a week and were tested for occult blood (trace 1 + to 4 +) and protein (trace 1 + to 4 +) through a standard semiquantitative test using the dipstick method (Multistix 10 SG, Siemens). The children having abnormal results in the initial screening were to be tested again 2 weeks later, while the children with positive results of both screening tests were to be asked to have routine urine tests. The Second Affiliated Hospital and Yuying Children’s Hospital of Wenzhou Medical University (WMU) was informed about the subjects with abnormal findings on the urine routine tests for further investigation and treatment. From February to December 2012, 12,497 school children were screened for urinalysis, and a long-term follow-up took place from December 2012 to March 2021 *via* an electronic medical record system that included hospitalization records, imaging studies, prescription medications, and outpatient clinic notes. Hematuria is defined as at least 5 red blood cells per high-power field. Proteinuria and nephrotic-range proteinuria were defined as urinary protein excretion ≥ 4 and ≥ 40 mg/h/m^2^, respectively. According to the group of hematuria and proteinuria, these children would be divided into three groups as the isolated hematuria (IH) group, the isolated proteinuria (IP) group, and the combined hematuria and proteinuria (CHP) group.

### Statistical Methods

All statistical analyses were performed using SPSS software version 26. Comparisons between groups were made using the chi-square and Fisher’s exact tests. The Bonferroni test was performed for pair-wise comparisons between groups. Results were considered significant with *p* ≤ 0.05.

## Results

### Results of Dipstick Screening

**FIGURE 1:**
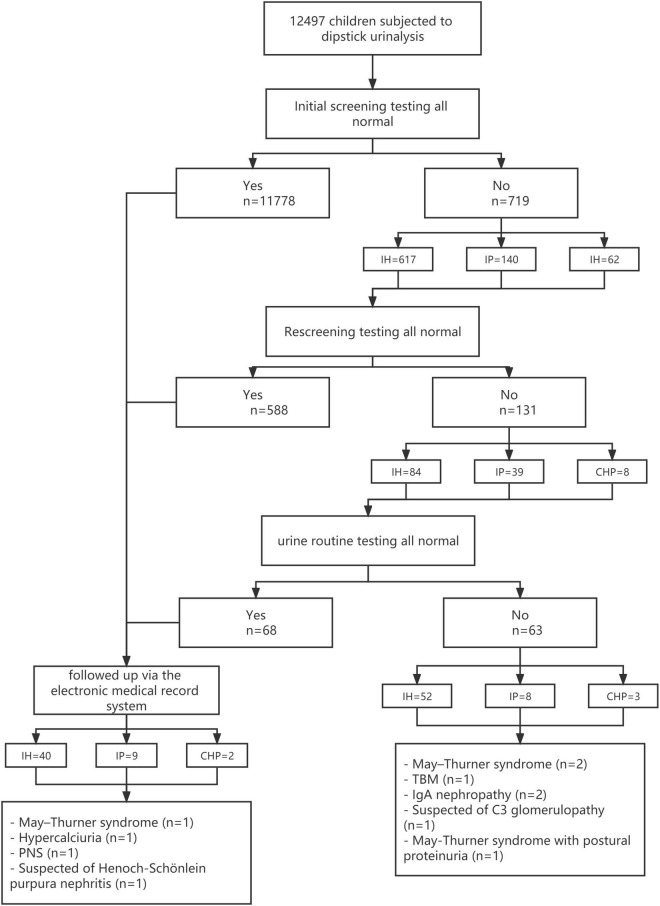
Screening of primary and secondary school children and their follow-up.

Figure 1 provides the results of the screening and follow-up. Table 1 provides the distribution of positive screening by grade. The detection rate was 5.75% at the first round of screening. No statistically significant difference was found in age distribution between groups. The detection rate was 36 (0.55%) among the primary school students and 95 (1.60%) among the secondary school students at the second round of screening. The difference in the detection rate was significant (*p* < 0.001), and the secondary school students were prone to having urine abnormalities than the primary school students.

**TABLE 1:** Distribution of positive screening by grade [n (%)].

Age group	Actual number of screenings	IH	IP	CHP	*p* value (primary and secondary students)	*P* value: IH vs. IP	*p* value: IH vs. CHP	*p* value: IP vs. CHP	*p* value: (positive detection rates)
**Initial screening**									
Primary	6550	178 (2.72)	33 (0.50)	23 (0.35)					
Secondary	5947	339 (5.70)	107 (1.80)	39 (0.66)	0.038				
**Rescreening**									
Primary	234	27 (11.54)	5 (2.14)	4 (1.71)					
Secondary	485	57 (11.75)	34 (7.01)	4 (0.82)	0.02*	0.069	1.000	0.099	<0.001*

*Fisher exact probability method; Pairwise comparisons were Bonferroni corrected; Numbers in the parenthesis indicate percentage.

Table 2 provides the distribution of positive screening by sex. The differences were statistically significant in different groups by sex (*p* < 0.001). The comparison of IH and IP groups in sex distribution was statistically significant (*p* < 0.001), and females were more likely to present IH (13.46%), whereas males generally showed IP (9.12%). There was no significant difference in the detection rate of the urine test according to sex (*p* = 0.419).

**TABLE 2:** Distribution of positive screening by sex [n (%)].

Sex	Actual number of screenings	IH	IP	CHP	*p* value (males and females)	*p* value: IH vs. IP	*p* value: IH vs. CHP	*p* value: IP vs. CHP	*p* value: (positive detection rates)
**Initial screening**									
Male	6863	216 (3.15)	100 (1.46)	24 (0.35)					
Female	5634	301 (5.34)	40 (0.71)	38 (0.67)	0.001				
**Rescreening**									
Male	340	33 (9.71)	31 (9.12)	3 (0.88)					
Female	379	51 (13.46)	8 (2.11)	5 (1.32)	< 0.001*	<0.001	1.000	0.083	0.419

*Fisher exact probability method; Pairwise comparisons were Bonferroni corrected; Numbers in the parenthesis indicate percentage.

### Re-evaluation and Final Diagnosis

Children suffering from urine abnormalities in both screening tests were asked to perform a further routine urine test. After the end of the urine screening, there were 63 children with abnormal urinalysis within 3 months. Among these children, there were 52 IH children including 2 children having the May–Thurner syndrome with renal ultrasound, and an 8-year-old girl who had urinary RBC (≥ 5/HPF in the urine sediment) many times. In addition, the RBC morphology of this girl in the urinary sediment indicated a bias toward the microhematuria derived from glomerular origin. The urine protein excretion was less than the normal index of 0.5 g/24 h, so she was diagnosed with TBM by renal biopsy. However, she was left untreated and was lost to follow-up right after her discharge from our hospital; 49 children were having continuous minor microscopic hematuria (RBC 5–10/HPF), but they had nothing abnormal by contrast phase microscopy and renal ultrasound and were advised to continue follow-up. Among these children, renal biopsy was performed on a 13-year-old girl during the follow-up period, and IgA nephropathy was diagnosed. The last follow-up of this girl was in August 2019, and the 24-h urinary protein and renal function fluctuations were kept within the normal range. There were 8 cases with IP which were followed up, and the quantification of their 24-h urine protein was less than 0.5 g/24 h, but all were lost to follow-up. The longest follow-up time was 16 months. During the follow-up period, there was a normal range of urine albumin and nothing abnormal was found by contrast phase microscopy and renal ultrasound. The 3 CHP children were found to have urinary RBC (≥ 5/HPF in the urine sediment) several times, and the morphology of the RBC in the urinary sediment indicated a bias toward hematuria derived from the glomerular origin with urine protein > 2 + many times. In addition, a 6-year-old girl from these 3 cases had been diagnosed with the May–Thurner syndrome and postural proteinuria, and until the last follow-up time in June 2018, the routine urine test and urinary microprotein of this girl were normal. The other 2 children of these 3 cases had persistent 24-h urinary protein quantification > 0.5 g/24 h, and in them, 12-year-old boy was pathologically diagnosed with IgA nephropathy, given steroids, and ACEI. The last follow-up was in September 2018, and the 24-h urinary protein excretion and renal function fluctuated within the normal range. A 12-year-old girl was suspected of C3 glomerulopathy and was lost to follow-up due to the patient’s transfer-out.

A total of 51 new-onset urinalysis abnormalities was collected after the screening period in our hospital for 9 years. Among them, 40 children presented with IH, 1 child was identified as having the May–Thurner syndrome with renal ultrasound, and 1 child was diagnosed with idiopathic hypercalciuria using medical examination. The rest of the children of continuous minor microscopic hematuria (RBC 5-10/HPF) nor nothing abnormal was found by contrast phase microscopy and renal ultrasound, and they were informed to continue follow-up. There were 9 children among this new onset of IP cases having 24-h urine protein quantification less than 0.5 g/24 h and no abnormal renal ultrasound, and they were still followed up. Among them, 2 children presented with new onset of CHP. One 14-year-old boy had a pathological diagnosis of primary nephrotic syndrome; the urinary protein once resolved with glucocorticoids; and was lost to follow-up in February 2015. The hematuria of 2 + to 3 + was found and 24-h urine protein quantification was detected for this boy. Another 9-year-old-boy was hospitalized for skin purpura and found to have urinary RBC (≥ 5/HPF in urine sediment) as well as urine protein > 2+ many times during hospitalization. Then, he was transferred to another hospital due to parental willingness, so the renal biopsy of this child was not performed at that time.

Over the 9-year-period after the end of the urine screening, a total of 114 follow-ups with incomplete electronic medical records was collected. There were 56 primary school-age children, and 6 of them had follow-ups longer than 3 years. Meanwhile, there were 58 secondary school-age children, and 6 children from them had follow-ups longer than 3 years. There was no significant difference in the proportion of the patients lost to follow-up between groups (X^2^ = 0.95, *p* > 0.05) (Table 3).

**TABLE 3:** Differences in lost to follow-up rates by grade [n].

	Follow-up time ≥ 3 years	Follow-up time<3 years	X^2^	*p* value
Primary	6	50		
Secondary	6	52	0.95	>0.05

### Cost of Various Screening Methods

Children were tested with urine dipsticks read manually in this mass screening. According to Table 4, the total cost of single detection was about $0.61 per person. Because the positive rate of the first screening was no more than 6%, the cost for screening twice was no more than $0.62 per person. And children who were found with urine abnormalities in both screening tests were invited for further routine urine test. So, the total cost of single detection of routine urine test was mainly composed of the cost of test ($3.00 per person) and the registration fee ($1.58 per person). The cost is up to $6.00 per person if the transportation fee is covered. In addition, the costs of urine dipsticks to read manually were considerably lower than a routine urine test.

**TABLE 4:** Cost composition of a single detection of urine dipstick.

Items		Screening cost ($)
Articles	Medical disposable latex gloves	0.03
	Urine cup and sterile tube	0.09
	Urine dipstick	0.25
	Photocopying (questionnaire)	0.08
Labor fee/time	Medical staff	0.16
Total cost		0.61

## Discussion

Chronic kidney disease (CKD) is a major public health problem in the world, and extensive epidemiological research in the adult population is available. However, little is known about the epidemiology of CKD in the pediatric population (1). Children with CKD face lifelong increase in morbidity and mortality as well as decreased quality of life (2). In Taiwan, the percentage of patients with heavy proteinuria decreased from 10.5% in 1992 to 7.1% in 1996 after adherence to urine screening (3). Moreover, the percentage of children requiring dialysis due to glomerulonephritis was decreased from 19 per million in 1992 to 8 per million in 1997 (2). In Japan, due to adherence to urine screening over several generations, the age of the people who developed into end stage renal disease (ESRD) has been rising year by year, and the number of new ESRD patients of lower than 20 years is less than that in America (4). As a result, if urine screening can be added to school health check-ups of primary or secondary school students in our region, more children with abnormal urinalysis results with no clinical manifestations can be found, which will contribute to enabling early detection of urine abnormalities and allowing for early treatment (5–8).

In Asia, Japan was the first country to start a national urinary screening program for school children on an annual basis in 1974. Between 1974 and 1986, the mean prevalence of hematuria or proteinuria on the second urine screen in the 6–11-year-old group was 0.65%, and the 12–14-year-old group was 1.39% (9). China has no national screening program, but studies have been successively carried out at the province and city levels. The school urinary screening program in Shanghai was conducted from 2003 to 2005, and more than 40,000 school children were examined, so the prevalence for hematuria or proteinuria was 1.00% based on the second urinary screening (7). Until then, no greater urine screening was ever undertaken in Wenzhou, China. Based on the results of this survey, the total prevalence for hematuria or proteinuria was 1.05%, and the detection rate for primary school children was 0.55%, while 1.60% for the secondary school children. According to the North American Pediatric Renal Trials and Collaborative Studies (NAPRTCS), congenital anomalies of the kidney and urinary tract (CAKUT) (48%) are the most important cause of CKD in children, and glomerulonephritis was (14%) the next most useful predictor, followed by hereditary nephropathies (10%) (1). Glomerulonephritis was the leading cause in children with the age of more than 12 years. IgA nephropathy was the most common type of primary glomerulonephritis (10, 11). There were 2 cases of IgA nephropathy, 1 case of TBM, 1 case of PNS, 1 case of the suspected purpura nephritis, and 1 case of the suspected C3 glomerulopathy collected in the 9-year medical record systems during the follow-up period despite the low rate of follow-up due to the parents’ lack of awareness about renal and urinary disorders.

Proteinuria is a common urinary abnormality in children, which can be used as an efficient indicator of early renal damage as well as for the progression of CKD to ESRD (12), and has not attracted much attention so far. The proportion of students with persistent proteinuria was 0.08% at the second screening for 6–year-olds and was increased to 0.37% for the 12–14-year-olds based on a 13-year follow-up in Japan (9). There was another Japanese study about the current progression status from the screening phase to the further investigation phase suggesting 5.9 and 23.6% of proteinuria-positive primary and high school students, respectively. Moreover, the number of students attending further investigation would decrease with increasing age (13). The prevalence of persistent proteinuria was 0.08% among primary school students and 0.57% among secondary school students. And 9 cases with new onset of IP were collected after the screening period of nine years. However, most of the children were lost to follow-up within 1 year, and could not provide prognostic information for persistent proteinuria. This may be linked to the lack of awareness of the significance of urinary abnormalities.

Currently, there are no specific ages for school urine screening. Given the CKD etiology of pediatrics, CAKUT predominated in younger patients, and glomerulonephritis was the leading cause in more than 12-year-old children (1). According to the research in Shanghai, Taiwan province, and Japan, the detection rate for urine abnormalities had increased with the increasing age (3, 7, 9), and there was a peak point at 12 years. Compared with primary school students, secondary school students were more likely to present with urine abnormalities. Given the study population, there was no significant difference in the proportion of patients lost to follow-up by age. Due to this, priority may be given to secondary school students under resource-constrained circumstances.

Among the countries that had performed urine screening programs, urine dipstick was chosen for its convenience, economic considerations, and good specificity (7, 9, 14). The cost of single detection using urine dipsticks was about $0.61 per person in this study, which was much lower than $3.05 (United States) (15). Because the examiners read the dipsticks in the school, the transportation fee was eliminated. In contrast, if all students choose to go to the hospital for a routine screening test, the cost of single detection of routine urine test is up to $6.00 per person, but this was not feasible in the large population size of the Chinese population.

In conclusion, there is a need to recommend school urinary screening programming as an item of school health examination for its convenience and economic considerations. Furthermore, we propose the secondary school-aged students as the screened population because of their higher detection rates. Considering most students were lost to follow-up in the third year after screening, we recommend urine screening every 3 years to ensure the efficacy of the school urinary screening programming.

## Data Availability Statement

The raw data supporting the conclusions of this article will be made available by the authors, without undue reservation.

## Ethics Statement

The studies involving human participants were reviewed and approved by Institutional Review Board of Children’s Hospital of Fudan University (study number 2012–174). Written informed consent to participate in this study was provided by the participants’ legal guardian/next of kin.

## Author Contributions

TC and QY made substantial contributions to acquisition, analysis, interpretation of data, and drafting the work. DW and XG contributed to protocol development, outcome assessment, and manuscript writing. JZ, HL, JY, and JL had primary responsibility for patient enrollment and outcome assessment and for writing the manuscript. HX, YG, and MC had substantial contributions to the conception and design of the work, analysis, interpretation of data, and revising the article for important intellectual content, and contributed to the final approval of the version to be submitted. All authors have read and approved the manuscript.

## Conflict of Interest

The authors declare that the research was conducted in the absence of any commercial or financial relationships that could be construed as a potential conflict of interest.

## Publisher’s Note

All claims expressed in this article are solely those of the authors and do not necessarily represent those of their affiliated organizations, or those of the publisher, the editors and the reviewers. Any product that may be evaluated in this article, or claim that may be made by its manufacturer, is not guaranteed or endorsed by the publisher.
